# Supplementary material for: Feasibility of Casein to Record Stable Isotopic Variation of Cow Milk in New Zealand
Source: Molecules. 2020 Aug 11;25(16):3658. doi: 10.3390/molecules25163658 (PMC7464366; doi:10.3390/molecules25163658)
Supplement: Supplementary file 1 [file molecules-25-03658-s001.pdf]

Supplementary

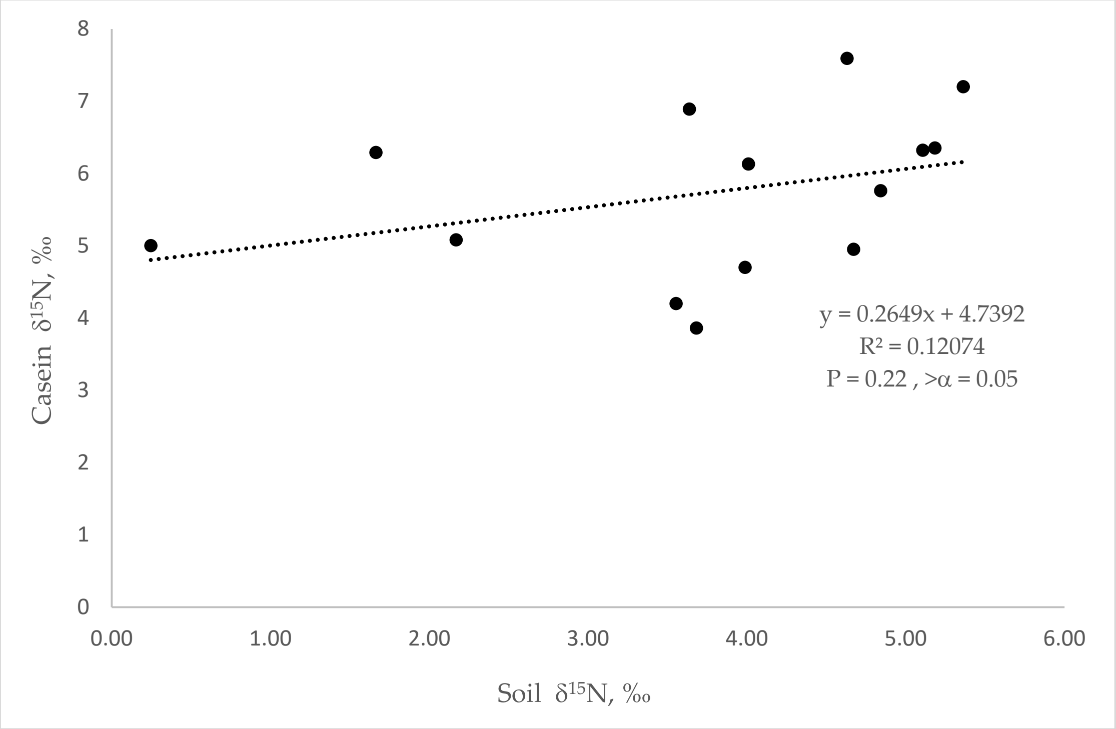

Figure S1. Casein $\delta^{15}N$ variation with Soil $\delta^{15}N$ that were obtained from the farming locations. It is demonstrated that there is only a weak correlation between casein $\delta^{15}N$ and soil $\delta^{15}N$ ($r = 0.34$, $p = 0.22$, $>\alpha = 0.05$).

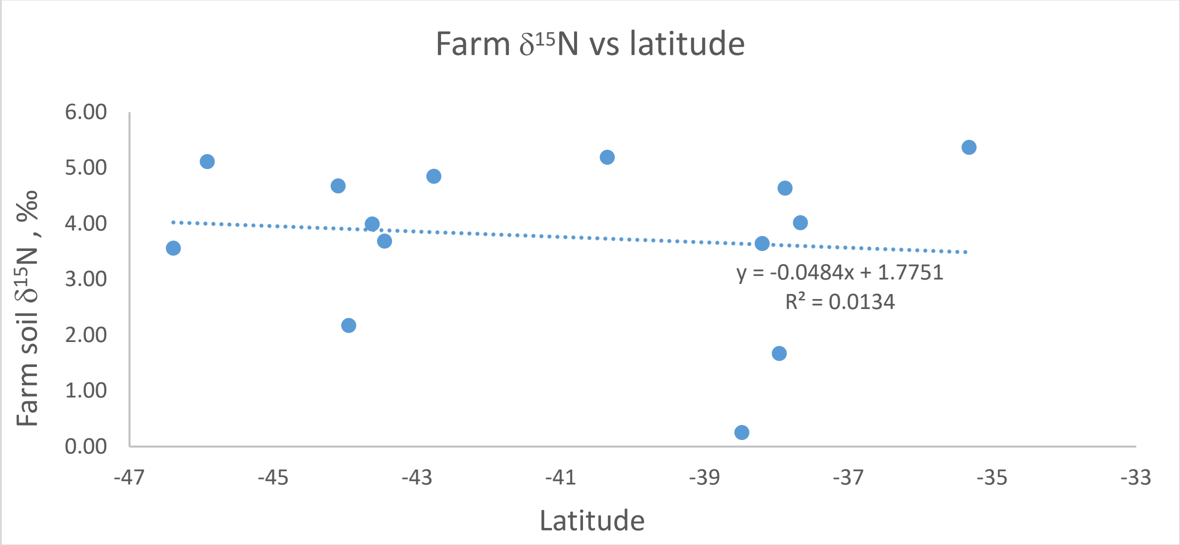

Figure S2. Farm soil $\delta^{15}N$ vs Latitude. Similarly, there is weak correlation between $\delta^{15}N$ and Latitude.

© 2020 by the authors. Submitted for possible open access publication under the terms and conditions of the Creative Commons Attribution (CC BY) license (http://creativecommons.org/licenses/by/4.0/).
